# Intravenous Vitamin C in Severe Sepsis: A Systematic Review of Evidence From 2020 to 2025

**DOI:** 10.7759/cureus.100867

**Published:** 2026-01-05

**Authors:** Rana Ahmed, Shashwat Shetty, Muhammad Qaiser Aziz Khan, Iqra Kalsoom, Shazia Qasim, Shair Bahadar Khan, Saad Abdullah

**Affiliations:** 1 Emergency Medicine, Hillingdon Hospitals NHS Foundation Trust, Uxbridge, GBR; 2 Orthopedics, Hillingdon Hospitals NHS Foundation Trust, Uxbridge, GBR; 3 Cardiac Surgery, Liaquat National Hospital, Karachi, PAK; 4 Medicine, Liaquat National Hospital, Karachi, PAK; 5 Medicine, Fatima Jinnah Medical University, Lahore, PAK; 6 General Medicine, Healthcare Polyclinic, Dammam, SAU; 7 General Medicine, Abbasi Shaheed Hospital, Karachi, PAK

**Keywords:** intravenous vitamin c, mortality, sepsis, septic shock, sofa score, systematic review, vasopressor

## Abstract

Sepsis is a life-threatening organ dysfunction caused by a dysregulated host response to infection, with high mortality despite advances in standard care. Intravenous (IV) vitamin C has been proposed as an adjunctive therapy due to its antioxidant, endothelial-stabilizing, and catecholamine-sparing properties, which may improve organ function and reduce vasopressor requirements. This systematic review evaluates evidence from 2020 to 2025 on IV vitamin C in adult patients with sepsis or septic shock. Five studies, including randomized controlled trials, cohort studies, and meta-analyses, were included. Results indicate modest physiological benefits, such as transient reductions in Sequential Organ Failure Assessment (SOFA) scores and vasopressor duration, but consistent improvement in mortality, ICU stay, or hospital length of stay remains unproven. Variability in outcomes is influenced by dosing regimens, timing of administration, patient severity, and cointerventions. Early administration, particularly within 6 h of sepsis recognition, may enhance physiological effects, while delayed therapy appears less effective. Adverse effects, including oxalate nephropathy, highlight the need for careful monitoring. Overall, IV vitamin C may serve as a supportive adjunct in sepsis management, but robust, large-scale trials are needed to clarify its clinical efficacy, optimal dosing, safety, and patient selection.

## Introduction and background

Sepsis is a complex and dysregulated host response to infection that results in life-threatening organ dysfunction and remains a major global health problem. According to the Global Burden of Disease, there were approximately 48.9 million cases of sepsis in 2017, resulting in nearly 11 million deaths [1]. Despite advances in antimicrobial therapy, fluid resuscitation, early goal-directed care, and organ-supportive interventions, mortality remains high, approximately 25-40% in severe sepsis and higher in patients with septic shock [2]. Current guideline-based management emphasizes early recognition, source control, hemodynamic stabilization, and timely antibiotic therapy, but no adjunctive pharmacologic therapy has consistently demonstrated a survival benefit [3].

Vitamin C (ascorbic acid) plays a critical role in multiple physiological processes and is markedly depleted during critical illness, including sepsis. Low plasma vitamin C levels are common in septic patients and are associated with increased organ dysfunction and mortality [4]. Vitamin C acts as a potent antioxidant, scavenging reactive oxygen species, and serves as a cofactor in catecholamine synthesis, endothelial stabilization, collagen formation, and immune regulation. These properties suggest that restoring adequate vitamin C levels might mitigate oxidative injury, improve endothelial and microvascular function, and enhance vasopressor responsiveness. High-dose intravenous vitamin C regimens frequently studied include 1.5 g every 6 h (total 6 g/day) or 50 mg/kg every 6 h, administered either as monotherapy or in combination with other therapies, such as hydrocortisone and thiamine [5].

Early pilot studies and smaller randomized controlled trials reported improved Sequential Organ Failure Assessment (SOFA) scores, reduced vasopressor requirements, and potential mortality benefits [6]. However, larger multicenter trials and meta-analyses published between 2020 and 2025 have produced inconsistent or neutral results. Recent meta-analyses of randomized controlled trials indicate that intravenous high-dose vitamin C does not significantly reduce short-term mortality but may reduce vasopressor duration and modestly improve SOFA scores at 72-96 h [7].

Considerable variability exists among studies in dosing regimens, timing of administration (early versus delayed), patient severity (sepsis versus septic shock), and use of cointerventions, contributing to inconsistent findings. Potential adverse effects, including oxalate nephropathy, highlight the importance of evaluating the safety and clinical benefit of IV vitamin C in sepsis. Given these uncertainties, a contemporary synthesis of high-quality evidence is needed. The primary aim of this systematic review was to evaluate the effect of intravenous vitamin C on mortality and SOFA score. Secondary aims included assessing its impact on vasopressor requirements, ICU and hospital length of stay, physiological outcomes, and treatment safety. By focusing on evidence published from 2020 to 2025, this review provides an updated and clinically relevant evaluation of IV vitamin C in sepsis management.

## Review

Materials and methods

Search Strategy

A systematic literature search was conducted in PubMed, Embase, Scopus, and the Cochrane Library for studies published between 2020 and 2025. Keywords included “sepsis,” “septic shock,” “intravenous vitamin C,” “ascorbic acid,” “mortality,” and “SOFA score,” using Boolean operators to combine terms and optimize retrieval. Additional hand searching of references cited in relevant reviews and meta-analyses was performed to ensure comprehensive coverage. Searches were limited to human studies published in English. All retrieved records were imported into a reference manager for duplicate removal and initial screening. Study selection and reporting were conducted in accordance with the Preferred Reporting Items for Systematic Reviews and Meta-Analyses (PRISMA) guidelines, and a flow diagram was used to document the identification, screening, eligibility assessment, and inclusion of studies [8].

Eligibility Criteria

Studies were included based on the PICO framework - Population: adult patients (≥18 years) with sepsis or septic shock, as defined by Sepsis-3 criteria, including both emergency department and ICU; Intervention: intravenous vitamin C administered as high-dose monotherapy or adjunctive therapy (1.5 g every 6 h, 2 g every 8 h, or weight-based dosing); Comparator: placebo or standard care without intravenous vitamin C; Outcomes: primary outcomes included mortality and SOFA score, secondary outcomes comprised vasopressor requirements, ICU and hospital length of stay, physiological improvements, and adverse events [9]. Eligible study designs included randomized controlled trials, prospective cohort studies, and systematic reviews/meta-analyses published between 2020 and 2025. Exclusion criteria encompassed animal studies, case reports, editorials, conference abstracts, and studies lacking extractable outcome data.

Study Selection

Two independent reviewers screened titles and abstracts for relevance, followed by full-text assessment against eligibility criteria. Disagreements were resolved through discussion or consultation with a senior investigator. A PRISMA flow diagram was used to document the selection process. Screening emphasized adherence to inclusion criteria while minimizing selection bias. Full transparency in study inclusion and exclusion was maintained throughout.

Data Extraction

Data were extracted independently by two reviewers using a standardized template. Extracted items included study characteristics (author, year, country, design), patient demographics, intervention details (dose, timing, duration), comparator arms, primary and secondary outcomes, and adverse events. When necessary, the corresponding authors were contacted to obtain missing data. Consistency between reviewers was confirmed prior to analysis.

Risk of Bias Assessment

The methodological quality of included studies was assessed using validated tools. Randomized trials were evaluated using the risk of bias 2 (RoB-2) tool [10], observational studies using risk of bias in non-randomized studies of interventions (ROBINS-I) [11], and systematic reviews/meta-analyses using a measurement tool to assess systematic reviews (AMSTAR-2) [12]. Domains assessed included randomization, allocation concealment, blinding, confounding, selective reporting, and completeness of outcome data. Studies were categorized as low, moderate, or high risk of bias.

Data Synthesis

Quantitative synthesis was performed when outcome homogeneity was present; otherwise, a qualitative summary was provided. Pooled analyses were calculated for mortality, SOFA score, and vasopressor duration when appropriate. Heterogeneity was assessed, and subgroup or sensitivity analyses explored differences by vitamin C dose, timing, patient severity, and cointerventions. Narrative synthesis complemented statistical findings for studies not amenable to pooling.

Results

Study Selection Process

**Figure 1:**
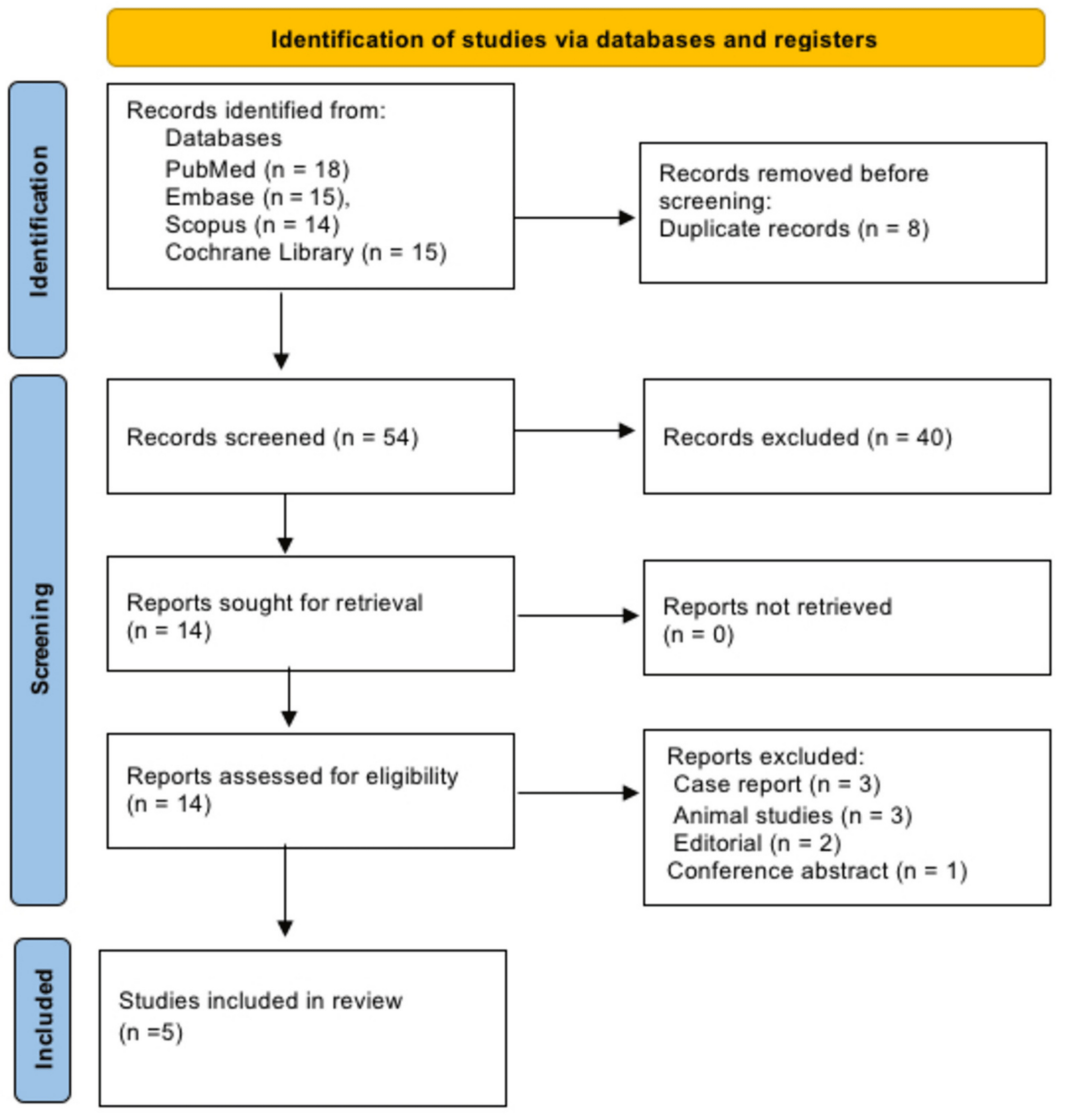
PRISMA 2020 flow diagram. PRISMA: Preferred Reporting Items for Systematic Reviews and Meta-Analyses

Figure 1 illustrates that a total of 62 records were identified through database searches, including PubMed (n=18), Embase (n=15), Scopus (n=14), and the Cochrane Library (n=15). After removing eight duplicate records, 54 studies were screened based on titles and abstracts, resulting in the exclusion of 40 irrelevant records. Fourteen full-text articles were assessed for eligibility, of which the following 10 were excluded: three case reports, three animal studies, two editorials, and one conference abstract. No reports were unable to be retrieved. Ultimately, five studies met all inclusion criteria and were included in the systematic review.

Characteristics of the Selected Studies

Table 1 summarizes the studies evaluating intravenous vitamin C in adults with sepsis or septic shock. Vandervelden et al. in their study randomized 292 emergency department patients to IV vitamin C (1.5 g every 6 h for four days) or placebo and found no significant difference in post-baseline SOFA scores, although patients with baseline SOFA scores ≥6 demonstrated modest improvement; mortality and ICU or hospital length of stay were unchanged [13]. Gonzalez-Vazquez et al. evaluated 34 critically ill ICU patients receiving adjunctive IV vitamin C and reported lower mortality and greater reductions in SOFA scores compared with standard care, without a significant effect on hospital length of stay [14]. Zeng et al., in a meta-analysis of eight randomized controlled trials, reported that high-dose IV vitamin C reduced short-term mortality in patients with sepsis but not in those with septic shock, and was associated with reduced vasopressor duration; however, no significant improvement in SOFA scores or ICU length of stay was observed [15]. Similarly, Sato et al. pooled multiple randomized trials using varying vitamin C regimens and demonstrated mixed effects on mortality, minimal impact on SOFA scores, modest reductions in vasopressor requirements, and largely unchanged ICU and hospital length of stay [16]. In contrast, the large multicenter Lessening Organ Dysfunction with Vitamin C (LOVIT) trial by Lamontagne et al. randomized 863 critically ill ICU patients with sepsis to high-dose IV vitamin C monotherapy (50 mg/kg every 6 h for 96 h) or placebo and reported a higher incidence of the composite outcome of death or persistent organ dysfunction at 28 days in the vitamin C group, with no improvement in mortality, SOFA scores, or length of stay, highlighting potential safety concerns associated with high-dose vitamin C monotherapy in this population [17].

**Table 1:** Characteristics of the selected studies. IV: intravenous; SOFA: Sequential Organ Failure Assessment; LOS: length of stay; ED: emergency department; ICU: intensive care unit; RCT: randomized controlled trial; LOVIT: Lessening Organ Dysfunction with Vitamin C

Studies	Population (adults with sepsis/septic shock)	Intervention (IV vitamin C)	Comparator	Primary outcomes	Physiological findings	Vitamin C dose	SOFA score/hospital stay
Vandervelden et al. (2025) [13]	292 adults presenting to the emergency department with sepsis or septic shock (Sepsis-3 criteria)	IV vitamin C 1.5 g every 6 h for 4 days (initiated within 6 h of presentation)	Placebo (normal saline)	No significant difference in average post-baseline SOFA score between groups	Sub-analysis showed greater SOFA improvement in patients with baseline SOFA ≥6; no mortality benefit observed	1.5 g every 6 h (total 6 g/day)	Avg. SOFA days 2-5: 1.98 versus 2.19 (NS); ICU and hospital LOS similar between groups
Gonzalez-Vazquez et al. (2024) [14]	34 critically ill adults with sepsis and SOFA ≥9 admitted to ICU	IV vitamin C as adjunctive therapy (added to standard care)	Standard care alone	Lower mortality in IV vitamin C group compared with control	Greater reduction in SOFA score over time compared with control; no significant difference in hospital LOS	2 g IV every 8 h	SOFA decreased more markedly in vitamin C group; hospital LOS unchanged
Zeng et al. (2023) [15]	Adults with sepsis±septic shock enrolled in randomized trials (2020-2022)	High-dose IV vitamin C (monotherapy)	Standard care or placebo	Decrease in short-term mortality only in sepsis subgroup, not septic shock	Reduced vasopressor duration; no significant improvement in ICU length of stay	Variable high-dose regimens	SOFA score at 96 h showed no significant difference
Sato et al. (2021) [16]	Adults with sepsis or septic shock across several randomized trials	IV vitamin C (various regimens including 1.5 g q6h or weight-based dosing)	Placebo/standard care	Mixed mortality results; no consistent survival benefit	Small reductions in vasopressor duration; minimal effect on SOFA	1.5 g q6h; or 50 mg/kg depending on study	ICU and hospital LOS are largely unchanged across pooled data
Lamontagne et al. (2022) [17]	863 critically ill adults with sepsis receiving vasopressor therapy in the ICU (LOVIT trial)	IV vitamin C monotherapy initiated within 24 h of ICU admission	Placebo (normal saline)	Composite outcome of death or persistent organ dysfunction at 28 days (higher in vitamin C group); no mortality benefit	No consistent improvement in organ dysfunction; signal toward increased persistent organ dysfunction in vitamin C group	50 mg/kg IV every 6 h for 96 h	No significant improvement in SOFA score; ICU and hospital LOS were similar between groups

Risk of Bias Assessment

Vandervelden et al. conducted a randomized, placebo-controlled trial and were rated low risk using RoB-2 due to appropriate randomization, blinding of participants and clinicians, intention-to-treat analysis, and objective outcome measurement [13]. Gonzalez-Vazquez et al. conducted a prospective cohort study and rated it as having serious risk according to ROBINS-I because of small sample size, non-randomized design, and potential confounding from baseline differences and cointerventions [14]. Zeng et al. conducted a meta-analysis of eight RCTs and rated the quality as moderate using AMSTAR-2, limited by heterogeneity in dosing and timing and by small trial sizes [15]. Sato et al. pooled multiple RCTs and the studies were rated low-to-moderate risk, with variability in trial quality and dosing regimens reducing confidence in mortality outcomes [16]. Lamontagne et al. (LOVIT trial) conducted a large multicenter randomized placebo-controlled trial and rated it low risk using RoB-2 due to robust randomization, blinding, prespecified primary outcomes, and intention-to-treat analysis, although interpretation of adverse events requires careful consideration [17]. These assessments are summarized in Table 2.

**Table 2:** Risk of bias assessment. RoB-2: risk of bias 2; ROBINS-I: risk of bias in non-randomized studies of interventions; AMSTAR-2: a measurement tool to assess systematic reviews; SOFA: Sequential Organ Failure Assessment; LOVIT: Lessening Organ Dysfunction with Vitamin C

Studies	Study design	Risk of bias tool	Risk of bias rating	Justification
Vandervelden et al. (2025) [13]	Randomized, placebo-controlled clinical trial	RoB-2 (Cochrane risk of bias 2)	Low risk	Randomized design with reported allocation concealment and blinding of participants, clinicians, and outcome assessors; prespecified primary outcome (post-baseline SOFA) and intention-to-treat analysis; low and balanced attrition between groups; outcome measurement objective (SOFA components, mortality). Minor concerns only if reporting of protocol deviations or selective reporting is unclear: overall trial conduct is robust.
Gonzalez-Vazquez et al. (2024) [14]	Prospective observational cohort	ROBINS-I	Serious risk	Non-randomized design with small sample (n=34) → high risk of confounding and imprecision. Likely selection bias (treatment allocation not random; clinicians may have preferentially given vitamin C to certain patients), unadjusted or inadequately adjusted analyses for baseline severity, and potential cointervention confounding (concurrent steroids/thiamine). Outcome measurement likely valid, but effect estimates at risk of bias due to confounding and small event counts.
Zeng et al. (2023) [15]	Systematic review and meta-analysis	AMSTAR-2	Moderate quality	Comprehensive synthesis of randomized trials with predefined inclusion criteria. Strengths: explicit search strategy and quantitative synthesis. Limitations: heterogeneity in interventions (dose, timing, cointerventions), clinical heterogeneity across trials, and some included trials with small size or risk of bias; not all subgroup/sensitivity analyses prespecified. Confidence in pooled estimates reduced, though methods are otherwise acceptable.
Sato et al. (2021) [16]	Meta-analyses of randomized trials (several publications)	AMSTAR-2	Low to moderate	Quality heterogeneous: some component RCTs were well-conducted (low RoB) while others had concerns (open-label, incomplete blinding, small sample sizes). Meta-analyses applying rigorous methods (prospective protocol, heterogeneity analyses, GRADE) rated higher; pooled estimates influenced by small trials and variable dosing regimens. Pooled evidence should be interpreted cautiously; strength of evidence moderate for vasopressor endpoints, low for mortality.
Lamontagne et al. (2022) [17]	Multicenter randomized, placebo-controlled clinical trial (LOVIT trial)	RoB-2 (Cochrane risk of bias 2)	Low risk	Large, multicenter randomized design with allocation concealment and blinding of patients, clinicians, and outcome assessors. Prespecified primary outcome (composite of death or persistent organ dysfunction at 28 days) and intention-to-treat analysis. Minimal missing data; outcomes measured objectively. High methodological rigor, though interpretation of adverse effects requires caution due to higher incidence of composite outcome in the intervention group.

Discussion

This systematic review evaluated evidence published between 2020 and 2025 regarding the use of intravenous (IV) vitamin C in adult patients with sepsis or septic shock. Overall, IV vitamin C appears to provide modest physiological benefits, including transient reductions in Sequential Organ Failure Assessment (SOFA) scores and vasopressor duration; however, consistent mortality benefit has not been demonstrated. In the multicenter randomized trial by Vandervelden et al., early administration of IV vitamin C did not significantly improve post-baseline SOFA scores or mortality compared with placebo. However, patients with higher baseline SOFA scores showed modest physiological improvement [13]. Gonzalez-Vazquez et al. reported reduced mortality and greater SOFA score reduction in a small cohort of critically ill ICU patients receiving adjunctive vitamin C, suggesting potential benefit in selected high-severity populations. However, these findings are limited by sample size and observational design [14]. Meta-analyses by Zeng et al. and Sato et al. further support that IV vitamin C may reduce vasopressor requirements but does not consistently improve SOFA scores, ICU or hospital length of stay, or mortality outcomes [15,16]. Importantly, the LOVIT trial by Lamontagne et al., a large multicenter randomized placebo-controlled study of 863 adults with sepsis in the ICU, found that high-dose IV vitamin C (50 mg/kg every 6 h for 96 h) did not significantly improve persistent organ dysfunction or 28-day mortality compared with placebo [17]. This trial, the largest to date, underscores the limited clinical benefit of vitamin C monotherapy in a high-severity ICU population and reinforces the need to consider patient selection, timing of therapy, and potential adverse effects when evaluating this intervention.

Heterogeneity across studies likely reflects differences in dosing strategies, timing of administration, baseline severity, and cointerventions. Regimens have included fixed high-dose protocols (e.g., 1.5 g every 6 h or 2 g every 8 h) and weight-based dosing (50 mg/kg every 6 h), administered either as monotherapy or in combination with other agents [18]. Evidence suggests that early administration, particularly within the first 6 h of sepsis recognition, may result in more pronounced physiological improvements, whereas delayed initiation is less effective [19]. Variability in patient populations from moderately ill emergency department patients to critically ill ICU patients with high baseline SOFA scores also contributes to inconsistent outcomes, as those with more severe organ dysfunction may respond differently to antioxidant therapy. Safety concerns, particularly regarding renal outcomes, have been highlighted in recent trials. High-dose vitamin C has been associated with hyperoxaluria and oxalate nephropathy, potentially leading to renal dysfunction in susceptible patients. These findings emphasize the importance of careful patient selection, avoidance of prolonged high-dose therapy in those with pre-existing renal impairment, and close renal monitoring during treatment.

Combination therapy, particularly vitamin C with thiamine and hydrocortisone (HAT protocol), has attracted interest as a potentially safer and more effective approach. Thiamine plays a critical role in mitochondrial energy metabolism and may reduce oxalate accumulation by promoting glyoxylate metabolism, potentially mitigating vitamin C-related renal toxicity. Thiamine and hydrocortisone have also been associated with reductions in vasopressor requirements and improvements in organ function in select patient populations. While combination therapy may enhance physiological effects and safety compared with vitamin C monotherapy, evidence for a mortality benefit remains inconclusive, and findings are inconsistent.

This review supports a cautious interpretation of IV vitamin C as an adjunctive therapy in sepsis. Although transient improvements in organ function and vasopressor dependence are encouraging, the evidence is limited by heterogeneous study designs, small sample sizes, inconsistent cointerventions, and variable reporting of adverse events. The lack of large, adequately powered trials targeting high-severity patients, as highlighted by the LOVIT trial, represents a critical knowledge gap [17]. Future research should prioritize multicenter trials with standardized dosing, early initiation, stratification by disease severity, and evaluation of combination therapies, long-term outcomes, renal safety, and cost-effectiveness. Biomarker-guided approaches may help identify patients most likely to benefit, further refining the role of IV vitamin C in contemporary sepsis management.

## Conclusions

Intravenous vitamin C in adult patients with sepsis or septic shock shows modest physiological benefits, including transient reductions in SOFA scores and vasopressor requirements, but consistent improvements in mortality, ICU stay, or hospital length of stay remain unproven. Variability in outcomes is largely driven by differences in dosing regimens (e.g., 1.5 g every 6 h, 2 g every 8 h, or 50 mg/kg every 6 h), timing of administration, baseline severity of illness, concomitant therapies such as corticosteroids and thiamine, and heterogeneity in study design. Early administration, particularly within the first 6 h of sepsis recognition, appears to maximize physiological effect, while delayed therapy may be less effective. Safety concerns, including oxalate nephropathy and hyperoxaluria, highlight the need for careful patient selection and monitoring. Overall, IV vitamin C may serve as an adjunctive therapy to transiently improve organ function and reduce vasopressor dependence. Still, robust evidence from large, multicenter, standardized trials is needed to define its true clinical efficacy, optimal dosing strategy, patient selection, and long-term outcomes in sepsis management.
